# Complete genome sequence of *Streptomyces diastaticus* strain GN02 and analysis of its biosynthetic gene clusters

**DOI:** 10.1128/mra.00258-26

**Published:** 2026-08-19

**Authors:** Janette González-Nava, Héctor Javier Elenes-Zazueta, Jorge Marcos-Viquez

**Affiliations:** 1 Department of Biological Systems, Universidad Autónoma Metropolitana, Unidad Xochimilco, Mexico City, Mexico; 2 Faculty of Chemical and Biological Sciences, Universidad Autónoma de Sinaloa 27774, Culiacán, Sinaloa, Mexico; 3 Department of Synthetic Biology, Openlabmx, Mexico City, Mexico; University of Pittsburgh School of Medicine, Pittsburgh, Pennsylvania, USA

**Keywords:** *Streptomyces*, biosynthetic gene clusters, complete genome sequence, actinobacteria, actinomycetoma, secondary metabolites

## Abstract

We report the first complete genome sequence of *Streptomyces diastaticus* strain GN02, isolated from a patient with actinomycetoma. The genome comprises a 6.8 Mb linear chromosome with a GC content of 73.5%. Genome analysis identified 23 biosynthetic gene clusters.

## ANNOUNCEMENT

The genus *Streptomyces* possesses genomes rich in biosynthetic gene clusters (BGCs) that produce bioactive secondary metabolites (1, 2). Although several species are opportunistic pathogens associated with actinomycetoma, no prior reports describe *Streptomyces diastaticus* isolated from human clinical samples, and complete genomes are unavailable (3). This study sequenced and characterized the genome of a clinical isolate to clarify its genetic diversity and secondary metabolite potential (Table 1), helping to consolidate *Streptomyces* as a model organism for the study of genetic regulation and the biosynthesis of compounds of biotechnological interest.

**TABLE 1:** Summary of the genomic and biosynthetic features of strain GN02

Feature	Result or value
Chromosome	
Identification	*Streptomyces diastaticus*
Genome length (bp)	6,816,733
No. of contigs	C1
ONT read N50 (bp)	4,816
Contig N50 (bp)	6,816,733
Contig L50	1
Chromosome type	Linear
GC content (%)	73.5
Genome coverage	37×
Genome quality	
Completeness	99.89%
Contamination	0.36%
No. of reads	81,489
Maximum read length (bp)	63,675
Total genes	5,746
Total of coding sequences (CDS)	5,497
No. of rRNAs	3
No. of transfer RNAs	53
Biosynthetic gene clusters (BGCs)	
No. of BGC regions	23
Nonribosomal peptide synthetase (NRPS)	6
Polyketide synthase (PKS)	1
NRPS/PKS hybrid	3
Terpene	5
Ribosomally synthesized and post-translationally modified peptides	5
Others	3

Strain GN02 was recovered from a clinical sample (grain culture) obtained from a patient diagnosed with actinomycetoma on the right leg. Bacterial growth was carried out in Difco brain and heart infusion agar at 37°C under aerobic conditions for four days, prepared according to the manufacturer’s exact instructions (BHI agar). Genomic DNA was extracted using the Wizard Genomic DNA Purification Kit (Promega) following the gram-positive bacteria protocol. Taxonomic pre-identification was achieved via PCR amplification of the 16S ribosomal RNA (rRNA) gene using universal primers 27F/1492R, under the conditions described (4). The 1,368-bp amplicon, sequenced by Plasmidsaurus, showed 99.4% identity to *S. diastaticus* NBRC-3714 (GenBank: NR_041209.1) via BLAST.

Whole-genome sequencing was performed by the Plasmidsaurus service using an Oxford Nanopore Technologies (ONT) MinION device. High-molecular-weight genomic DNA underwent standard magnetic bead purification to eliminate short fragments (<3 kb) and enrich for long reads, without an additional explicit size-selection step. An amplification-free long-read library was constructed via transposome complexes (tagmentation) using ONT v14 chemistry. Sequencing was conducted using a primer-free protocol on R10.4.1 flow cells. Basecalling was performed using the Dorado Super Accurate v5.2 model (5), applying default raw filtering with a quality threshold of *Q*-score ≥ 10, automated adapter trimming, and the exclusion of reads < 300 bp. To refine quality control, Filtlong v0.2.1 (6) retained the top 95% highest-quality reads. Genome size and downsampling were estimated using Autocycler v0.5.2 (7). *De novo* assembly was executed in parallel with Flye v2.9.6+ (8) and Hifiasm v0.25.0 (9), while Plassembler v1.8.0+ (10) was employed for assembly. The resulting assemblies were refined with Autocycler and polished using Medaka v1.8.0 (11). Assembly completeness, contamination, and quality metrics were evaluated using CheckM v1.2.2 (12). Genomic taxonomy was confirmed using Sourmash v4.6.1 (13) and Mash v2.3 (14) based on genomic similarity against the Genome Taxonomy Database. Finally, annotation was carried out using the National Center for Biotechnology Information Prokaryotic Genome Annotation Pipeline v6.10 (15), and metabolic pathways of BGC regions were classified with antiSMASH v8.0.4 (16). Default parameters were used for all software.

This first complete genome of *S. diastaticus* enriches taxonomic and evolutionary knowledge outside its soil niche, opening new avenues to explore specialized secondary metabolites.

## Data Availability

The data sets generated during this study are available in the NCBI databases under BioProject PRJNA1422247, BioSample SAMN55303835, Sequence Read Archive raw reads SRR38062489, Assembly GCA_055232075.1, and Whole Genome Sequencing project JBUYFG000000000.1.
